# The combined effect of lifestyle intervention and antioxidant therapy on sperm DNA fragmentation and seminal oxidative stress in IVF patients: a pilot study

**DOI:** 10.1590/S1677-5538.IBJU.2021.0604

**Published:** 2021-08-30

**Authors:** Peter Humaidan, Thor Haahr, Betina Boel Povlsen, Louise Kofod, Rita Jakubcionyte Laursen, Birgit Alsbjerg, Helle Olesen Elbaek, Sandro C. Esteves

**Affiliations:** 1 Aarhus University Department of Clinical Medicine Denmark Department of Clinical Medicine, Aarhus University, Denmark; 2 Skive Regional Hospital The Fertility Clinic Skive Denmark The Fertility Clinic Skive, Skive Regional Hospital, Denmark; 3 Regional Hospital Herning Department of Obstetrics and Gynecology Denmark Department of Obstetrics and Gynecology, Regional Hospital Herning, Denmark; 4 ANDROFERT Clínica de Andrologia e Reprodução Humana Campinas SP Brasil ANDROFERT, Clínica de Andrologia e Reprodução Humana, Campinas, SP, Brasil; 5 Universidade Estadual de Campinas Faculdade de Ciências Médicas Departamento de Cirurgia Campinas SP Brasil Departamento de Cirurgia, Divisão de Urologia, Faculdade de Ciências Médicas, Universidade Estadual de Campinas (UNICAMP), Campinas, SP, Brasil

**Keywords:** DNA Fragmentation, Reproductive Techniques, Assisted, Infertility

## Abstract

**Purpose:**

Sperm DNA fragmentation (SDF) and seminal oxidative stress are emerging measurable factors in male factor infertility, which interventions could potentially reduce. We evaluated (i) the impact of lifestyle changes combined with oral antioxidant intake on sperm DNA fragmentation index (DFI) and static oxidation-reduction potential (sORP), and (ii) the correlation between DFI and sORP.

**Materials and Methods:**

We conducted a prospective study involving 93 infertile males with a history of failed IVF/ICSI. Ten healthy male volunteers served as controls. Semen analysis was carried out according to 2010 WHO manual, whereas seminal sORP was measured using the MiOXSYS platform. SDF was assessed by sperm chromatin structure assay. Participants with DFI >15% underwent a three-month lifestyle intervention program, primarily based on diet and exercise, combined with oral antioxidant therapy using multivitamins, coenzyme Q10, omega-3, and oligo-elements. We assessed changes in semen parameters, DFI, and sORP, and compared DFI results to those of volunteers obtained two weeks apart. Spearman rank correlation tests were computed for sORP and DFI results.

**Results:**

Thirty-eight (40.8%) patients had DFI >15%, of whom 31 participated in the intervention program. A significant decrease in median DFI from 25.8% to 18.0% was seen after the intervention (P <0.0001). The mean DFI decrease was 7.2% (95% CI: 4.8-9.5%; P <0.0001), whereas it was 0.42% (95%CI; -4.8 to 5.6%) in volunteers (P <0.00001). No differences were observed in sperm parameters and sORP. Based on paired sORP and DFI data from 86 patients, no correlation was observed between sORP and DFI values (rho=0.03).

**Conclusion:**

A 3-month lifestyle intervention program combined with antioxidant therapy reduced DFI in infertile men with elevated SDF and a history of failed IVF/ICSI. A personalized lifestyle and antioxidant intervention could improve fertility of subfertile couples through a reduction in DFI, albeit controlled trials evaluating reproductive outcomes are needed before firm conclusions can be made.

Trial registration number and date: clinicaltrials.gov NCT03898752, April 2, 2019.

## INTRODUCTION

Male infertility accounts for approximately 20-30% of infertility cases; however, combined with female factor infertility it contributes to at least 50% of infertility cases (1). Male infertility involves a broad causative spectrum, including infections, endocrine disorders, cryptorchidism, genetic conditions, and varicocele, but in up to 50% of cases, the cause of male infertility remains unknown (2). An important contributor to male factor infertility is sperm DNA fragmentation, which is generally not routinely assessed before or during infertility treatment (3, 4).

It has been shown that more than 20% of normozoospermic males have a DNA fragmentation index (DFI) >27%, resulting in decreased chances of conception after intrauterine insemination (IUI) (5). Moreover, in vitro fertilization (IVF) success rates are lower in men with high DFI (6, 7). A recent meta-analysis comparing IVF and/or intracytoplasmic sperm injection (ICSI) success rates in men with low and high DFI reported higher clinical pregnancy rates in favor of the low DFI group, regardless of fertilization method; for IVF, the odds ratio (OR) was 1.92 (95% CI; 1.33-2.77) and for ICSI, 1.49 (95% CI; 1.11-2.01) (8).

Notably, a routine semen analysis with semen parameters within normal ranges does not preclude the presence of elevated DNA fragmentation, which has been associated with poor reproductive outcome after IUI and IVF treatment (3, 4, 6). In addition, it has been suggested that the use of testicular sperm to be superior as compared to ejaculated sperm for ICSI in patients with DFI levels >29% (9, 10), and that the use of this individualized approach resulted in increased live birth rates -especially for patients in whom previous ICSI cycles using ejaculated sperm had failed (3, 6). Since testicular sperm aspiration is an invasive procedure, other less-invasive interventions that could potentially reduce DFI levels in high DFI males should be explored.

Oxidative stress (OS) is a potentially modifiable cause of DFI elevation, seen in male factor infertility (11, 12). A surrogate measure for the level of seminal OS is the oxidation-reduction potential (ORP). The ORP can be quantified by using the automated Male Infertility Oxidative System (MiOXSYS) (13). This automated method has been suggested as a less time-consuming and more efficient way of assessing the complete redox balance of all known and unknown oxidants and antioxidants in semen (14). However, until now, results are conflicting regarding the correlation between the seminal ORP level, routine semen parameters, and DFI (15–17). Moreover, to suggest a clinical implementation of ORP measurement and DFI testing in daily practice, a potential causal inference needs to be made.

To further explore this emerging field of male infertility, the objectives of the present study were to evaluate the combined impact of a three-month questionnaire-based personal lifestyle change and oral antioxidant therapy on DFI and ORP in men with infertility and DFI levels >15%, undergoing medically assisted reproduction (MAR); moreover, the possible correlation between DFI and ORP was also investigated.

## MATERIALS AND METHODS

### Ethics

The Central Denmark Region Committees on Health Research Ethics approved the conduct of this study, file number 1-10-72-148-19. The study was registered at clinicaltrials.gov: NCT03898752. All participants provided written informed consent.

### Participants

In this pilot study, a total of 93 consecutive men with idiopathic or unexplained infertility undergoing MAR treatment with their partners were prospectively included between November 2018 and May 2020. Only patients who had at least two failed IVF/ICSI trials for no apparent reasons, resulting in either embryo developmental arrest, implantation failure, biochemical pregnancy, or miscarriage were invited to participate. Before embarking on our IVF program, males underwent a standard workup, including history, physical examination, semen analysis, and serology. Hormone testing, ultrasound scan, and genetic testing were applied as appropriate.

Participants were non-smokers, including no intake of cannabis, and were not taking medication with known gonadotoxic effects when entering the IVF program. Moreover, at the first consultation, all patients were advised by the treating physician to reduce exposure to pesticides, irradiation, and excess heat in daily life and work. Furthermore, patients with sperm concentrations below 5 million/mL in raw semen had a testicular ultrasound to exclude malignancies. Genetic testing including karyotype, Y-chromosome microdeletion screening, and cystic fibrosis transmembrane conductance regulator (CFTR) gene analysis were performed in males with sperm concentrations below 1 million/mL in the raw semen, according to the recommendation by the Danish Health Authority. Patients with varicocele, malignancies, genetic causes of infertility, and those using any medication were excluded from the study.

### Healthy volunteers

Through direct contact to the local military base a convenience sample consisting of 10 healthy male conscript volunteers with a mean age of 31 years, defined as non-smokers and presumably not suffering from any known disease served as controls. None of the controls had children or fathered previous pregnancies. The main aim of this group was to investigate the biological variability in semen parameters. The controls delivered two semen samples, respecting a 14-day interval under similar circumstances as the IVF patients, regarding abstinence time and examination method in the same laboratory. These samples were blinded, and hence, the DFI laboratory could not identify the male volunteers, nor did the DFI laboratory know the results of the first DFI test. The first measurements of sORP and standard semen quality parameters were also blinded for the laboratory technician who performed the analysis of the second test.

### Sampling and semen analysis

Patients were instructed to have a minimum of two days and a maximum of three days abstinence before delivering the semen sample on-site. Semen analysis was performed as part of diagnostic testing according to WHO 2010 criteria (18), measuring ejaculate volume, sperm concentration, total sperm number, total motility, and progressive motility.

### ORP measurement

ORP in semen was measured in-house using the MiOXSYS platform (MiOXSYS, Aytu BioScience Inc, Englewood, CO, USA). MiOXSYS is a qualitative electrochemical test for the detection of oxidative stress in fresh or frozen semen specimens. A 30μL of the semen sample was loaded into the MiOXSYS sensor, which was immediately inserted into the analyzer. The analysis was completed in approximately three minutes, and the final static oxidation-reduction potential (sORP) reading in millivolt (mV) was displayed on the MiOXSYS display screen. This value was normalized to the sperm concentration of the sample. The result was reported as mV/10^6^ sperm/mL. A cut-off value of 1.36 mV/10^6^ sperm/mL sORP has previously been proposed based on correlations to sperm concentration, motility, and sperm morphology (14). sORP values above the normal range imply an imbalance between oxidants and antioxidants, signaling the presence of oxidative stress in the specimen. No positive/negative control protocols are provided by the manufacturer. However, to assess test reproducibility, we conducted paired assessments, using specimens of ten patients. Variability was analyzed by calculating the mean difference between the two readings. The mean difference was 0.10 mV/10^6^ sperm/mL sORP (Standard error: 0.20; P=0.60).

### DFI analysis

Part of the semen sample was shipped to a central laboratory (SPZ lab A/S, Copenhagen, Denmark) for DFI analysis. An aliquot of 0.2mL of the semen sample was transferred to a cryo-vial and diluted with 0.2mL sperm washing medium (G-IVF™ PLUS, Vitrolife) and snap-frozen at -80°C. The samples were shipped in a dry-shipper container containing liquid nitrogen (-196°C). DFI was determined using the sperm chromatin structure assay (SCSA) (19). Results were expressed as the percentage of sperm with DNA fragmentation (% DFI) using the flow cytometer software. A minimum of 10,000 events was recorded, and all tests were run in parallel with positive and negative controls.

### Questionnaire-based personalized lifestyle intervention

All males with a DFI >15% were asked to embark on a three-month lifestyle intervention program based on a questionnaire consisting of lifestyle parameters. The patients received individualized recommendations as to lifestyle intervention according to their answers to a structured questionnaire including a total 68 of questions, covering among others smoking, alcohol consumption, caffein intake, diet, exercise, body weight, stress, and work-related exposure (Appendix). The proposed lifestyle interventions included primarily general health recommendations for adults from the Danish Health Authority (www.sst.dk/da/Viden). In brief, at least 30 minutes of exercise daily was recommended. Regarding nutrition and food consumption, a reduction in meat intake (maximum of 500g/week) and, in particular, caution about red meat, more intake of fruit and vegetables, and reducing high sugar-containing drinks, including soda and energy drinks were recommended. Moderate alcohol intake <6 units per week was also recommended. All patients embarking on our IVF program were already non-smokers by recommendation. We considered any potential work-related exposures and recommended, for example, facemasks to reduce airborne exposure when relevant. In case of a subjective feeling of stress, we recommended mindfulness. Finally, we advised against having the cellphone in the trouser pocket and the laptop on the lap.

The decision to use DFI >15% as cut-off was based on (i) our preliminary SCSA data, indicating that reproductive outcomes of couples undergoing IVF/ICSI are reduced when thresholds exceed 15% (20), and (ii) clinical SCSA data on natural pregnancy published elsewhere (21), revealing that men producing ejaculates with adequate routine semen parameters and less than 15% DFI had no delay in pregnancy initiation (<4 months), whereas counterparts with 15%-30% DFI took a longer time to achieve pregnancy (4-12 months). The decision to use a three-month lifestyle intervention period was based on the time for spermatogenesis, previously reported to be 64+/-8 days (22).

### Antioxidant therapy

All patients with DFI >15% received antioxidant therapy, which included a combination of daily nutraceutical supply of coQ10 100mg (OmniQ10 Energy, Biosym A/S), one multivitamin (Apovit®) tablet, and 1g Omega-3 (Apovit®) daily. One multivitamin tablet contained: vitamin A 800ug, vitamin B1 1.1mg, vitamin B2 1.4g, vitamin B6 1.4mg, vitamin B12 2.5ug, niacin 16mg, pantothenacid 6mg, folic acid 200ug, vitamin C 80mg, vitamin D 5ug, vitamin E 12mg, magnesium 100mg, molybdenum 50ug, iron 14mg, zinc 10mg, copper 1mg, manganese 2mg, chromium 40ug, selenium 55ug, and iodine 150ug. The choice of the above commercially available multivitamin tablet was primarily based on patient convenience as it is widely available in our market and the fact that the manufacturer's recommended daily doses was mostly in accordance to the recommended dietary allowance. Moreover, published data indicate that the above vitamin/mineral combination and dosage provides adequate antioxidant effects (23).

### Follow-up and outcome

After three months of intervention (lifestyle and antioxidant therapy), a new semen sample was delivered, respecting the same abstinence time as for the initial semen sample. The treating physician checked patient compliance with the intervention at the follow-up visit. The primary outcome was the possible effect of life-style intervention and antioxidant therapy on sORP and DFI levels. Other outcomes were to investigate a possible correlation between sORP and DFI and compare the effect of the intervention on elevated DFI in IVF patients to the biological DFI variation in healthy controls.

### Sample size

A formal sample size calculation was not possible as this was a single-arm exploratory pilot study using for the first time a personalized lifestyle intervention in combination with antioxidant therapy and a DFI cut-off of 15%. However, a post-hoc power analysis was conducted on the basis of the mean difference in DFI results from specimens of healthy volunteers obtained two weeks apart and the results of studied patients from before to after intervention.

### Statistical methods

Baseline data is described with median and range. Spearman rank correlation test was computed for sORP and DFI results to have a nonparametric comparator to the regression model. The linear regression model was based on the standard assumptions, plots of the residuals, and a leverage plot to investigate extreme outliers. In case data did not meet the assumptions on the original scale, we investigated log-transformed values. We applied a chi-square test to evaluate the association between DFI and sORP as a binary variable using the cut-offs stated. Adjustment for total motility was made by multiplying the motility percentage on the sORP value. For the follow-up analysis pre/post-intervention, we used paired t-test on the differences after testing distribution with quantile-quantile plots and variance in Bland-Altman plots. If data did not meet the assumptions on the original scale, log-transformed values were investigated, reporting the median ratio between pre/post-intervention measurements. The estimates are given with the 95% confidence interval (CI) and the minimum and maximum values when relevant.

In order to compare healthy volunteers and IVF patients with follow-up data, we used a one-sample t-test on the differences before and after intervention in IVF patients and the mean in controls. Sensitivity analyses were also made according to the 95% CI around the mean in the controls. The rationale in these analyses was to compare biological variation in semen parameters to those seen after the intervention. One patient lost to follow-up was not included in the analysis. A total of eight men decided to take another antioxidant therapy than recommended by the unit (Punalpin®; N=4) and Inoman®; N=4)). These patients were included in the primary analysis but excluded from the sensitivity analysis. Data were analyzed using STATA IC©, version 16.1, StataCorp LLC, USA.

## RESULTS

### Lifestyle and antioxidant intervention and impact on DFI, sORP on semen parameters

**Figure 1:**
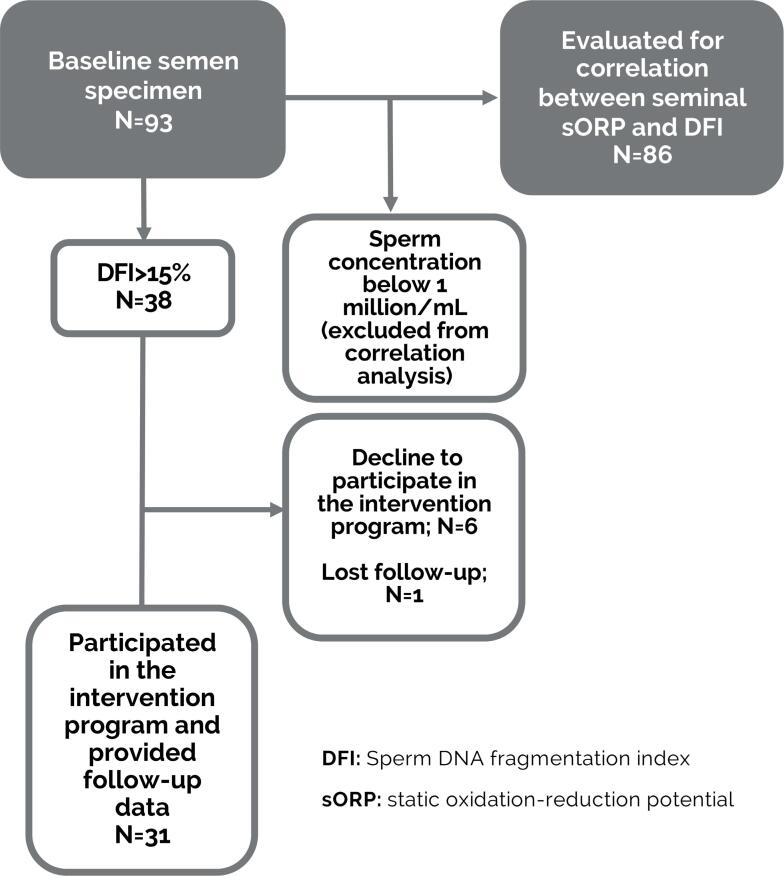
Flow diagram showing total patient breakdown.

A total of 38 of 93 patients (40.8%) with a history of failed IVF had DFI >15% (Figure-1). Of them, six males declined to participate in the three-month intervention despite having DFI >15% (DFI ranged from 15.4-17.5% in those six patients). In addition, one male was lost to follow-up. Thus, a total of 79% (31/38) of patients with DFI >15% participated in the three-month personalized lifestyle intervention, after which a follow-up semen sample was delivered.

**Figure 2:**
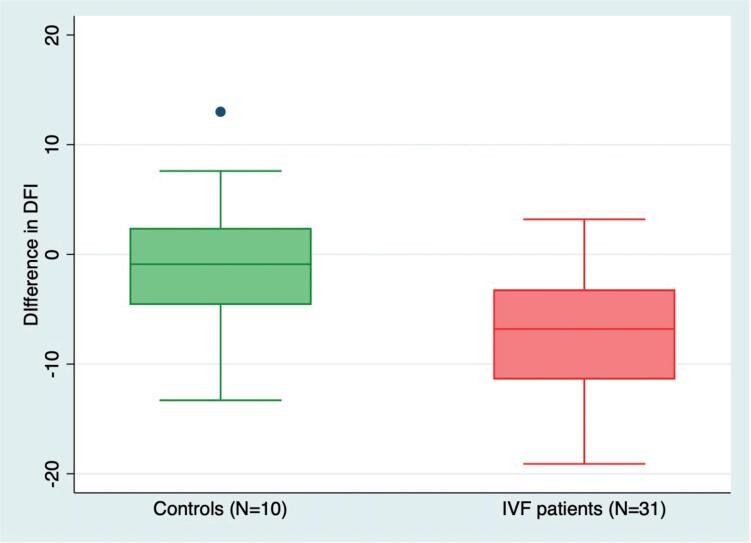
Boxplots showing the difference between sperm DNA fragmentation (DFI) measurements of 10 healthy controls who delivered two semen specimens within a 14-day interval and 31 IVF patients with baseline DFI >15% who had lifestyle intervention and antioxidant therapy for 3 months. The boxplot includes the median (horizontal line in the box), 25-75% interquartile range box (i.e., representing 50% of the data), minimum and maximum values excluding outliers (whiskers extending outside of the box), and outlier (blue dot). The DFI differences between the groups were significant (P <0.0001).

Table-1 shows the patient's DFI, sORP, and semen parameters, before and after a 3-month lifestyle intervention and antioxidant therapy. A significant decrease in median DFI from 25.8% to 18.0% was seen after the intervention (P <0.0001). The mean DFI decrease was 7.2% (95% CI: 4.8-9.5%; P <0.0001), and its correspondent distribution ranged from -19.7% to 5.3%, i.e., at the individual level, a high variance was observed as some males experienced a large decrease in DFI, whereas others had a slight increase in DFI (Figure-2). Notably, a total of 84% (26/31) had a decrease in DFI, and 29% (9/31) had a DFI of ≤15% at follow-up. In our study, a total of 13 men had baseline DFI above 27%; after the intervention, 69% (9/13) of them had a DFI below 27%. Only one patient discontinued the interventional program before completion for unknown reasons. All others complied with the intervention program for three months.

**Table 1:** Semen parameters, sperm DNA fragmentation index (DFI) and seminal static oxidation-reduction potential (sORP) after 3-month lifestyle intervention and antioxidant therapy in 31 infertile men with DFI>15%.

	Baseline	Follow-up*	Ratio**	P-value
Sperm concentration (x10^6^/mL)	12.9 (7.0-23.7)	13.4 (7.3-24.4)	1.03 (0.8-1.4)	0.812
Total motile sperm count (x10^6^/mL)	15.5 (8.1-29.9)	13.7 (7.6-24.7)	0.89 (0.6-1.3)	0.499
%DFI	25.8 (23.0-29.0)	18.0 (15.1-21.6)	0.70 (0.6-0.8)	0.000
sORP (mV/10^6^ sperm/mL)	1.3 (0.7- 2.5)	0.9 (0.4-1.8)	0.67 (0.4-1.1)	0.114

* After 3-month intervention program

Values are median and interquartile range

** Ratio between post-intervention and baseline values

A total of 28 males were available for sORP analysis before and after lifestyle intervention as three patients had a semen concentration below 1 million/mL. According to the manufacturer's instructions, sORP should only be performed in specimens with sperm concentration above 1 million/mL. No statistically significant sORP reduction was seen after lifestyle intervention as given by the median ratio between pre/post lifestyle intervention (sORP values: 0.67; 95% CI: 0.41 to 1.11). The distribution of sORP values was large; some men experienced an 8-fold increase in sORP post-intervention, whereas others had a 20-fold reduction in sORP (range: 0.05 to 8.10). In the subgroup of patients with sORP >1.36mV/10^6^ sperm/mL (N=12), we observed a significantly lower sORP in the second measurement than the first measurement (t-test: median ratio 0.34; 95% CI: 0.15 to 0.77), but the equivalent non-parametric test did not reveal a significant difference. Sensitivity analysis excluding patients who administered either Punalpin (N=4) or Inoman (N=4) yielded similar results (data not shown).

The median ratio in sperm concentration before and after the intervention was small and not statistically significant (median ratio 1.03; 95% CI: 0.8 to 1.4). The total motile sperm count was also not statistically different from before to after intervention (median ratio 0.89; 95% CI: 0.6 to 1.3).

### Correlation between DFI and sORP at baseline

A total of 86 patients from the 93 total study population was eligible for the correlation analysis (Table-2). Seven patients with a sperm concentration in the raw sample less than 1 million/mL were excluded from the correlation analysis, but not from the intervention part of the study. The seven patients with <1 million/mL were clear outliers, presenting with extremely high sORP measurements assumed to be caused by the normalization to very low sperm concentration. Using the sORP cut-off at 1.36 and the DFI cut-off at 15% to evaluate outcome as binary variables, no correlation between sORP and DFI was seen (OR=0.81; 95% CI; 0.34 to 1.90, Table-3).

**Table 2:** Baseline data of 86 infertile men included in the correlation analysis between sperm DNA fragmentation index (DFI) and seminal static oxidation reduction potential (sORP).

Variable		Median (IQR)
**Demographics:**		
	Age; years	35 (24-55)
	Body Mass Index (BMI); kg/m^2^	26 (19-42)
**Semen analysis:**		
	Volume; mL	2.4 (0.5-8.0)
	Concentration; 10^6^/mL	25.0 (1.0-300.0)
	Total motile count; 10^6^	32.4 (0.4-556.0)
**Sperm function:**		
	DNA Fragmentation Index (DFI); %	14.3 (4.0-74.8)
	Seminal Oxidation Reduction Potential (sORP)*; mV/10^6^ sperm/mL	1.30 (0.00-30.50)

* Two samples had sORP values which were below 0.00 at the MIYOXIS display –these were relabeled as 0.00.

IQR: interquartile range

**Table 3:** 2x2 table of dichotomized sperm DNA fragmentation index (DFI) and static oxidation-reduction potential (sORP).

	Normal DFI: ≤15% (N)	High DFI: >15% (N)	Total (N)
Normal sORP level: ≤1.36 mV/10^6^ sperm/mL (N)	24	22	46
High sORP level: >1.36 mV/10^6^ sperm/mL (N)	23	17	40
Total (N)	47	39	86

The chi-square statistic is 0.24. The *P* value is 0.62.

**Figure 3:**
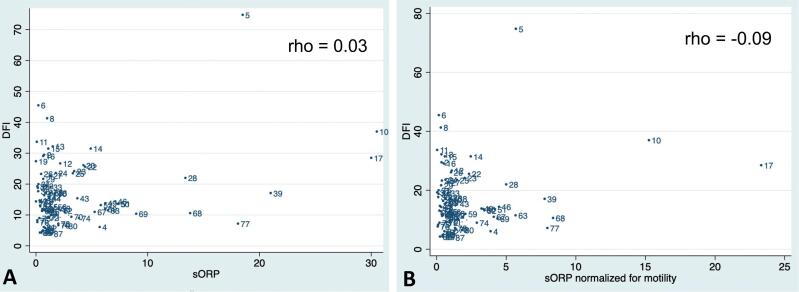
Scatterplot of the correlation between Sperm DNA Fragmentation and Seminal Oxidation Reduction Potential, excluding patients with sperm concentration below 1 million/mL. A total of 86 patients was included. As raw data did not meet the assumptions to fit a linear model, a Spearman's rank correlation was run to test the overall hypothesis of monotonic correlation between sORP and DFI (A), and sORP normalized for motility and DFI (B). The test indicated a very weak correlation: rho=0.03 and -0.09, respectively.

As raw data did not meet the assumptions to fit a linear model, a Spearman's rank correlation was used to test the overall hypothesis of monotonic correlation between sORP and DFI. Again, the test did not support any correlation (rho=0.03; Figure-3A). After log-transformation, data passed the standard assumptions of the linear regression model. However, the 95% CI of the regression coefficient exceeded 0 (0.05; 95% CI; -0.04 to 0.15), and consequently, there was no evidence to support an association between DFI and sORP. A multiple linear regression model adjusted for age and BMI showed a similar regression coefficient of 0.05 (95% CI; -0.04 to 0.15). As a recent study suggested that the sORP adjusted for total motility might be a better outcome marker (24), this was also investigated; however, with similar results (rho= -0.09; Figure-3B).

### Lifestyle intervention compared to biological variation in DFI

In the volunteer group (N=10), the mean difference between the two consecutive DFI measurements performed 14 days apart was 0.42% (95% CI: -4.8 to 5.6%), indicating no systematic difference between one DFI measurement to the other. Moreover, the DFI difference in volunteers was significantly lower than the relatively high difference observed after lifestyle and antioxidant intervention in IVF patients (P <0.00001; Figure-2). The sensitivity analyses comparing mean DFI difference in IVF patients according to the 95% CI of the mean difference in controls was also highly significant (P <0.00001). The differences in sORP from the first to the second measurement after 14 days were not normally distributed and had unequal variance. The median sORP difference was 0.12 lower at the second measurement after 14 days, but ranged from 15.91 lower to 2.95 higher (P=0.15).

A post-hoc power analysis based on the mean DFI results ultimately obtained from the two specimens of volunteers and patients subjected to the intervention indicated that the power of the studied cohort to have an 80% chance of detecting, as significant at the 5% level, a decrease of approximately 7% in DFI with the interventional program was 96.5%.

## DISCUSSION

### Main findings

This pilot study investigated (i) the effect of lifestyle and antioxidant therapy on DFI and ORP in a single-arm intervention trial in male IVF patients with IVF failure and DFI >15%, (ii) the correlation between sORP and DFI, and (iii) lifestyle intervention compared to biological variation in DFI.

We found that a three-month lifestyle changes alongside oral antioxidant intake reduced DFI, whereas no significant effect was seen on sperm concentration and total motile sperm count. The DFI reduction seen in the IVF patients was significantly higher than the DFI biological variation in the control group of healthy volunteers who delivered a semen sample 14 days apart without lifestyle intervention. Additionally, no significant effect of lifestyle intervention and antioxidant treatment was observed on sORP; however, in the subgroup of patients with elevated sORP >1.36 (N=12), we observed a significantly lower sORP by parametric testing, albeit this was not significant in the nonparametric test. Thus, this finding warrants further exploration in a larger prospective trial. Finally, no significant correlation between sORP and DFI was seen.

### Interpretation

The present study was not designed to evaluate sORP or DFI as individual markers of reproductive health, and thus, the results cannot be used as evidence for one marker being superior to the other. In contrast, our interpretation of the lack of correlation between sORP and DFI is that these two qualitative seminal markers (at least when measured as reported herein) should be considered separately, i.e., an elevated sORP does not necessarily correlate with an elevated DFI and vice versa. Thus, although oxidative stress is widely reported to be one of the major causes of elevated DFI (25, 26), oxidative stress, when measured by sORP, cannot be directly correlated to DFI.

In relation to this aspect, a recent retrospective cross-sectional study also investigated the correlation between DFI and sORP in male infertility patients (N=1068) (24). Similar to our study, the conclusion was that the DFI and sORP should be considered as two separate measurements. However, in contrast to the present study, the authors did not exclude a potential correlation between DFI and sORP when taking into account sperm motility in the sORP measurements. Nevertheless, the baseline characteristics of the patients in the aforementioned study differ remarkably from this study as those patients generally had higher DFI and sORP levels. Moreover, DFI was measured differently (i.e., using the Halosperm G2 test kit), and importantly, it could be argued that the linear relationship assumed by receiver operating characteristics curves, made in that study, cannot sufficiently estimate cut-points for optimal sensitivity and specificity in exponential sORP data.

Intervention-based studies for elevated DFI and sORP in male infertility only recently started to emerge. One study investigated the effect of a three-month administration of an antioxidant preparation containing a variety of vitamins and nutrients. The study included a group of patients with male infertility of idiopathic (N=119) or unexplained (N=29) origins (12). That study evaluated the effect of antioxidant therapy on DFI and sORP, reporting that antioxidant treatment significantly improved both DFI and sORP in both patients subgroups. This effect was even more pronounced when evaluating the group of men with high baseline DFI -in that study defined as DFI >30% and using the sperm chromatin dispersion test. Another recent study included men from a single fertility center based on DFI >16% (N=35), investigating the effect of intervention with 1800mg freeze-dried açai pulp per day (27). After at least 74 days of follow-up, a statistically significant and relatively high reduction in DFI (-17.03±2.51%) was observed. In contrast, a reasonably large and well-conducted RCT reported that antioxidant intervention did not reduce DFI generally or in the group of males with a high baseline DFI >25% (28).

The most recent Cochrane meta-analysis concluded that the current evidence on intervention with antioxidants in subfertile men is inconclusive due to the low quality of the evidence (29). Nevertheless, in the Cochrane meta-analysis, low-quality evidence according to GRADE anticipates increased live birth rates in case of antioxidant intervention; thus, there is a need for more well-conducted studies in this field. Collectively, results of the antioxidant intervention remain equivocal, and interstudy comparison is restricted by differences in baseline characteristics, the intervention per se, and differences in methods to evaluate the outcome. In this aspect, it could be speculated whether regulation should be improved in the field of antioxidants, as frequently antioxidants are considered food supplements -not medical drugs- leading to inadequate rigor regarding efficacy and safety.

Importantly, not only lifestyle interventions and antioxidants seem to impact the DFI (30). A meta-analysis evaluating the effect of follicle-stimulating hormone (FSH) therapy on DFI in male infertility reported a significant reduction in the mean DFI after treatment with exogenous FSH (4.24%; 95% CI 0.24-8.25%) (31). Also, varicocele repair has been shown to significantly reduce DFI in infertile men with palpable varicocele (32, 33). In these lines, use of testicular sperm for ICSI has been explored in non-azoospermic males with high DFI values, as DFI was shown to be markedly reduced in testicular as compared to ejaculated sperm (9, 34).

### Lifestyle and semen quality

To the best of our knowledge, this is the first study evaluating the effect of lifestyle intervention combined with antioxidant therapy on DFI, ORP and standard semen parameters. Although the effect of the present lifestyle intervention cannot lead to isolated evidence for any single intervention, we point to the fact that the lifestyle interventions used were quite basic and with only minor adjustments to the general health recommendations for adults set by the Danish Health Authority. It could be speculated that most males already follow these simple recommendations, but in fact, a recent large (N=1149) questionnaire-based study in subfertile men provided evidence against this assumption (35). Interestingly, subfertile men were reported to more frequently have lifestyle risks and on top of that, being poorly educated on lifestyle factors and their association with semen quality.

The evidence for intervention with physical exercise is mostly circumstantial. Cross-sectional studies show that a sedentary lifestyle is associated with low semen quality (36), whereas self-reported increased physical activity appears to be associated with increased motility (37) and total sperm count (38). Cell-phone “trousers” storage has been associated with increased DFI (39, 40). This effect -among others- could be mediated by radiation damage to the NAD+ isocitrate dehydrogenase enzyme (41). As for diet, a recent large cross-sectional study in young Danish men (N=2935) found that a prudent diet -as recommended in the present study- was associated with higher median sperm count as compared to a more “western” diet, including high amounts of processed foods, high on fat and sugar (42).

### Strengths and limitations

The limitations of the present pilot study are the small sample size, lack of formal a priori power calculation, and lack of a control group of male infertility patients with DFI >15%. However, our single-arm trial was primarily designed to obtain preliminary evidence for the efficacy of the interventional treatment. Thus, the present findings could be considered pivotal for the design and power calculation of future trials. In these lines, our post-hoc analysis based on the mean DFI results obtained from volunteers and treated patients indicated that the number of included subjects was close to that needed to obtain 100% power, using type I and type II errors of 0.05 and 0.20, respectively, to detect a decrease of approximately 7% in DFI with the interventional program.

A potential selection bias is the fact that patients who declined to participate in the follow-up (N=6) were all close to the DFI threshold. In total, only six males with a DFI between 15-20% at baseline were part of the study. Another limitation refers to the use of only DFI and ORP as outcome measures. Other indicators of oxidative stress such as lipid peroxidation, reactive oxygen species assessment, 8-oxoguanine determination could have provided additional information, but these assays were not available to us.

A strength of the study is that we selected patients based on previous IVF failure and baseline DFI values shown to affect the probability of pregnancy potentially. The inclusion of a healthy volunteer group for comparison is also a strength of the study as random biological variations occurring within 14 days do not appear to be the cause of the DFI reduction observed after three month's lifestyle intervention in the IVF patient group. We did not evaluate reproductive outcomes in this pilot study; however, natural pregnancies during the intervention were reported by two patients. Whether the DFI reduction after an intervention like ours translates into improved reproductive outcomes, both naturally and after ART, warrants further research. Nevertheless, recent data indicate that the magnitude of SDF decrease after other interventions (e.g., microsurgical varicocelectomy) was similar to that reported in the current study, with a potential positive impact of on reproductive success (32).

## CONCLUSIONS

We herein report that a three-month lifestyle change and antioxidant therapy program significantly reduced the DFI. This effect was statistically significant compared to a healthy control group who delivered two semen samples 14 days apart without any lifestyle intervention. No difference in ORP was seen after lifestyle intervention. Moreover, no correlation between sORP measured by MiOXSYS and DFI measured by the SCSA method was observed. This finding suggests that these two seminal markers should be evaluated separately. Personalized lifestyle and antioxidant intervention may potentially improve the fertility of subfertile couples through an improved DFI, albeit larger controlled trials evaluating pregnancy and live birth rates are needed before firm conclusions can be made.

## APPENDIX

### Baseline questionnaire

A large number of factors, including lifestyle can lead to reduced fertility in men. Recent research within the field shows that males can improve their fertility by lifestyle intervention.

At the Regional Hospital in Skive, a trial is being carried out, in which we investigate different factors impacting male fertility. We already have a presumption about some of the important factors, but we want to document the effect in depth. Thus, your participation in this study is important to move the field ahead.

In the questionnaire, please provide information about your life and lifestyle as accurately as possible. Based on the information, we will subsequently review your answers and suggest personalized life style changes wherever possible. The goal is for you to possibly achieve an improvement in fertility prior to embarking on fertility treatment.

In connection with the study, your sperm **quantity** will be examined. Moreover, an advanced **quality** analysis of the sperm (e.g., DNA fragmentation analysis) will also be performed. As the production of new sperms takes approximately 3 months, possible changes in quantity and quality will be assessed 3 months after lifestyle intervention through a repeat sperm analysis.

**Identification:**

1. Name: ___________________________________________________________________
2. CPR: ____________________________________________________________________
3. Email: ___________________________________________________________________
  **Information on fertility and infertility**
4. **Have you achieved pregnancy / children in previous relationships?**
  If yes, enter the year your last child was born: _____________
5. **Have you achieved pregnancy / children in the current relationship?**
  If yes, enter the year your last child was born: _____________
  **Information about genetic disorders in you or your family**
6. **Are you a carrier of a hereditary disease or a gene defect?**
  If yes, state which hereditary disease and / or gene defect: _______________________________________
7. **Is there anyone in your family who is a carrier of a hereditary disease or a gene defect?**
  If so, state which hereditary disease or gene defect and who in the family has this disease: ___________________________________________
  **Diseases and medication consumption**
8. **Have you previously been admitted to hospital or psychiatric ward for treatment?**
  If yes, please state the following:
    **Table:**
    Year	Name disease
  Possibly, Additional Information:
  _______________________________________________________________
  _______________________________________________________________
9. **Do you take any kind of medicine?**
  If yes, please state preparation/medicine, dose and against which disorder:
  _______________________________________________________________
  _______________________________________________________________
  _______________________________________________________________
  _______________________________________________________________
  _______________________________________________________________
10. **Did you take any medication during the last 6 months?**
  If so, please indicate the preparation/medicine and for which illness:
  _______________________________________________________________
  _______________________________________________________________
  _______________________________________________________________
  _______________________________________________________________
  _______________________________________________________________
11. **Are there any medications you cannot tolerate?**
  If yes, please indicate preparation/medicine:
  _______________________________________________________________
  _______________________________________________________________
  _______________________________________________________________
  _______________________________________________________________
  _______________________________________________________________
12. **Do you have malformations? (for example, were you born with both testicles in your scrotum?). All types of malformations must be mentioned:**
  Supplementary comments: ____________________________________________
  _______________________________________________________________
  _______________________________________________________________
  _______________________________________________________________
  _______________________________________________________________
13. **Do you have or have you had varicose veins in your scrotum? (pouches, varicocele)**
14. **If so, have you had surgery for varicose veins in the scrotum?**
15. **Have your testicles / scrotum at any time been exposed to an impact, injury, inflammation or alike?**
  Supplementary comments: __________________________________________
16. **Do you have any kind of chronic diseases? (eg: diabetes, high blood pressure or similar)**
  If so, which chronic disease (s): ________________________________________
  _______________________________________________________________
  _______________________________________________________________
  _______________________________________________________________
  _______________________________________________________________
17. **Do you suffer from the following (put x in the correct answer for each disorder)?**
  **Table:**
  Asthma	Supplementary comments: ___________________________
  Allergy	Supplementary comments: ___________________________
  Hay fever	Supplementary comments: ___________________________

18. **Do you have problems with upset stomach, abdominal pain or flatulence?**
  Do you suspect that consumption of specific foods cause these nuisances?:
  Yes □ Gluten
  Yes □ Milk
  Yes □ Eggs
  Yes □ What food types? ____________________________________________
  _______________________________________________________________
  _______________________________________________________________
19. **Have you had any type of cancer?**
  Which type of cancer: ___________________________
  Year this cancer was discovered? _________________
20. **Did you receive chemotherapy or radiation to treat cancer?**
  What type of chemotherapy / radiation? : ___________________________________________
21. **Have you had any acute illnesses during the last 6 months?**
  Which disease/illness?: _________________________________________________________
  _______________________________________________________________
  _______________________________________________________________
  _______________________________________________________________
  _______________________________________________________________
22. **Have you had a fever above 38 ° C for more than 24 hours during the last 6 months?**
23. **How many days of fever? ______________________________________**

24. **Have you had mumps (parotiditis) as a child?**
25. **How old were you when you had mumps? ______________**

26. **Have you been vaccinated against mumps?**
  **Information on general well-being**
27. **For each statement in the following form, please tick the option which comes closest to how you have felt during the last 2 weeks?**
  **Table:**
    During the last 2 week	All the time	Most of the time	A little more than half the time	A little less than half the time	A little of the time	At no time
    I have been happy and in good mood						
    I have feltcalm and relaxed						
    I have felt active and energetic						
    I woke up fresh and rested						
    my everyday life has been filledwith things that have interestedme						
  **Information about diet, lifestyle, etc.**
28. **Is your diet mixed or vegetarian?**
  Answers include all types of meat (red meat, poultry, seafood, etc.)
  □ Vegetarian. I never eat meat.
  □ Mixed. I eat meat approx. 3 times a week.
  □ Mixed. I eat meat at least 6 times a week.
29. **Intake of meat from poultry.**
  I eat poultry about______ times a month.
30. **Intake of meat from seafood.**
  I eat seafood about ______ times per month.
31. **How much meat do you eat approximately per meal?**
  □ I eat 100 grams or less
  □ I eat about 250 grams
  □ I eat 200 grams or more
32. **Do you take a multi-vitamin pill every day or other types of vitamin preparations?**
  If yes, please indicate which preparation(s) and how long you have been taking it/(them:
  Preparation: ____________________ Number of months: ___________________________
  Preparation: ____________________ Number of months: ___________________________
  Preparation: ____________________ Number of months: ___________________________
  Preparation: ____________________ Number of months: ___________________________
  Preparation: ____________________ Number of months: ___________________________
33. **Do you use dietary supplements - for example with protein?**
  If yes, state preparation for how long you have been taking supplements (months):
  Preparation: ____________________ Number of months: ___________________________
  Preparation: ____________________ Number of months: ___________________________
  Preparation: ____________________ Number of months: ___________________________
  Preparation: ____________________ Number of months: ___________________________
  Preparation: ____________________ Number of months: ___________________________
34. **Do you prefer light or dark bread?**
  □ I never eat dark bread
  □ I eat mostly light bread.
  □ I eat both light and dark bread.
  □ I eat mostly dark bread.
  □ I never eat light bread.
35. **How many times a day do you eat vegetables or fruit?**
  Number of times per day: ____________________
  (e.g. an apple, a carrot or other vegetables / fruit corresponding to about 100 grams (e.g. salad, etc.)).
36. **How often do you drink coffee?**
  □ I do not drink coffee
  □ I rarely drink coffee
  □ I drink coffee once a day
  □ I drink coffee several times a day
37. **Approximate number of cups of coffee per day: _____________________**

38. **How often do you drink tea?**
  □ I do not drink tea
  □ I rarely drink tea
  □ I drink tea once a day
  □ I drink tea several times a day
39. **Approximate number of cups of tea per day: ____________________**

40. **How often do you drink sugary soft drinks such as sodas, juices, energy drinks, juices and the like?**
  □ I rarely drink that kind of thing
  □ I drink it once a day
  □ I drink it several times a day.
41. **Approximate number of glasses per day: ___________________**

42. **How often do you eat chocolate, cakes and sweets?**
  □ Very often (several times a day)
  □ Once a day.
  □ Occasionally (3 times a week)
  □ Rarely (once a week)
  □ Never
43. **Your height and weight**
  Weight ___________ kg
  Height ___________ cm
44. **How many items of alcohol do you drink per week?**
  ________ items of beer.
  ________ items of wine.
  ________ items of spirits.
  Information for calculating the number of units:
  1 regular beer = 1 item
  1 strong beer = 1.5 items
  1 glass of wine = 1 item
  4 cl spirits = 1 item
  1 “galco-pop” = 1.5 items (eg Barcardi Breezer)
45. **Smoking habits:**
  □ I have never smoked
  □ I smoke about ____ cigarettes (or pipe tobacco) daily.
  □ I smoke e-cigarettes.
  □ I have stopped smoking.
46. **When did you stop smoking?**
  □ During the last 3 months.
  □ 3 to 6 months ago.
  □ 6 to 12 months ago.
  □ Over 1 year ago.
47. **I used to smoke about _________ cigarettes (or pipe tobacco) daily and have smoked for about ___ years.**

48. **Do you use snus?**
  □ I have never used snus
  □ I have used snus about ______ times a day
  □ I have stopped using snus
  If you use snus, state which brand: ______ Number of months you have taken it: ______________
49. **How many times a week do you exercise? ___________**

50. **Approximate duration per time ___________ min.**

51. **How big a part of your training is cardio training (swimming, cycling, running, etc.)?**
  □ Approx. 0% (I only do yoga, strength training, etc.)
  □ Approx. 25%
  □ Approx. 50%
  □ Approx. 75%.
  □ 100%.
52. **Did you ever used performance-enhancing drugs? (e.g. anabolic steroids)**
  Specify preparation: ___________________________________
  Number of months you took it: __________
53. **Do you consider yourself to be in good shape?**
  □ Yes
  □ No.
  □ Do not know
54. **What type of bath do you take?**
  □ I always take a shower
  □ I occasionally use the bath or spa. (approx. once a month)
  □ I often use a bath or spa. (at least once a week).
  **Work environment**
55. **What is your position / profession? __________________________________**
  Which of the following descriptions apply to your work environment?
56. **Does your job involve handling toxic substances such as chemicals and pesticides? (e.g.: hairdresser, farmer or similar)**
  Supplementary comments: ____________________________________________
57. **Does your work involve a risk of metal intake? (e.g. welders, plumbers and the like)**
  Supplementary comments: _______________________________________
58. **Does your job involve exposure to air pollution (e.g. exhaust fumes) or inhaling dust (for example when cutting or grinding)?**
  Supplementary comments: _______________________________________
59. **Is your work stressful?**
  □ Yes, to a greater extent
  □ Yes, to a lesser extent
  □ Yes, at times
  □ No.
60. **Does your work take place in a warm environment? (for example: chef, baker and the like).**
  Supplementary comments: ____________________________________________
61. **Does your work involve wearing protective clothing/overalls that can increase your body temperature making you sweat often during light work? Please note whether this outfit also includes trousers, which can increase the temperature in the groin.**
  □ Yes, I often sweat at work
  □ Yes, the protective clothing also includes trousers
  □ No.
62. **Do you sit down for more than 4 hours a day? (eg: office work, driver).**
  If yes, indicate the number of hours per day you sit down: ________________
63. **Do you often sit with your laptop on your lap?**
64. **Does your job involve a lot of driving? (for example: salesman, driver, etc).**
  If yes, indicate the number of hours per day you drive: __________
65. **Do you use heat in the car seat?**
66. **Do you often travel by plane? (e.g.: business man or similar).**
  If so, how many times a month do you travel by plane?: _______
67. **Do you often have your mobile phone in your trouser pocket?**
68. **Does your work mean that you often eat lunch or dinner in connection with work/business?**
  □ Yes, we have a lunch club or similar in connection with work
  □ Yes, I often eat evening meals in connection with work
  □ No, I rarely eat lunch or dinner in connection with work
  **You have reached the end of the questionnaire**
  **Thank you for your participation**

## References

[1] Agarwal A, Mulgund A, Hamada A, Chyatte MR. A unique view on male infertility around the globe. Reprod Biol Endocrinol. 2015; 13:37. 10.1186/s12958-015-0032-1 PMC4424520 25928197

[2] De Kretser DM, Baker HW. Infertility in men: recent advances and continuing controversies. J Clin Endocrinol Metab. 1999; 84:3443-50. 10.1210/jcem.84.10.6101 10522977

[3] Esteves SC, Zini A, Coward RM. Best urological practices on testing and management of infertile men with abnormal sperm DNA fragmentation levels: the SFRAG guidelines. Int Braz J Urol. 2021; 47: 1250-8. 10.1590/S1677-5538.IBJU.2020.1004 PMC8486448 33566471

[4] Esteves SC. Are specialized sperm function tests clinically useful in planning assisted reproductive technology? Int Braz J Urol. 2020; 46:116-23. 10.1590/S1677-5538.IBJU.2020.01.03 PMC6968890 31851468

[5] Bungum M, Humaidan P, Spano M, Jepson K, Bungum L, Giwercman A. The predictive value of sperm chromatin structure assay (SCSA) parameters for the outcome of intrauterine insemination, IVF and ICSI. Hum Reprod. 2004; 19:1401-8. 10.1093/humrep/deh280 15117894

[6] Esteves SC, Zini A, Coward RM, Evenson DP, Gosálvez J, Lewis SEM, et al. Sperm DNA fragmentation testing: Summary evidence and clinical practice recommendations. Andrologia. 2021; 53:e13874. 10.1111/and.13874 PMC7988559 33108829

[7] Bungum M, Humaidan P, Axmon A, Spano M, Bungum L, Erenpreiss J, et al. Sperm DNA integrity assessment in prediction of assisted reproduction technology outcome. Hum Reprod. 2007; 22:174-9. 10.1093/humrep/del326 16921163

[8] Simon L, Zini A, Dyachenko A, Ciampi A, Carrell DT. A systematic review and meta-analysis to determine the effect of sperm DNA damage on in vitro fertilization and intracytoplasmic sperm injection outcome. Asian J Androl. 2017; 19:80-90. 10.4103/1008-682X.182822 PMC5227680 27345006

[9] Esteves SC. Testicular versus ejaculated sperm should be used for intracytoplasmic sperm injection (ICSI) in cases of infertility associated with sperm DNA fragmentation | Opinion: Yes. Int Braz J Urol. 2018; 44:667-75. 10.1590/S1677-5538.IBJU.2018.04.03 PMC6092652 30020584

[10] Esteves SC, Roque M, Bradley CK, Garrido N. Reproductive outcomes of testicular versus ejaculated sperm for intracytoplasmic sperm injection among men with high levels of DNA fragmentation in semen: systematic review and meta-analysis. Fertil Steril. 2017; 108:456-467.e1. 10.1016/j.fertnstert.2017.06.018 28865546

[11] Agarwal A, Panner Selvam MK, Arafa M, Okada H, Homa S, Killeen A, et al. Multi-center evaluation of oxidation-reduction potential by the MiOXSYS in males with abnormal semen. Asian J Androl. 2019; 21:565-9. 10.4103/aja.aja_5_19 PMC6859659 31006711

[12] Arafa M, Agarwal A, Majzoub A, Panner Selvam MK, Baskaran S, Henkel R, et al. Efficacy of Antioxidant Supplementation on Conventional and Advanced Sperm Function Tests in Patients with Idiopathic Male Infertility. Antioxidants (Basel). 2020; 9:219. 10.3390/antiox9030219 PMC7139646 32155908

[13] Agarwal A, Sharma R, Roychoudhury S, Du Plessis S, Sabanegh E. MiOXSYS: a novel method of measuring oxidation reduction potential in semen and seminal plasma. Fertil Steril. 2016 Sep 1;106(3):566-573.e10. 10.1016/j.fertnstert.2016.05.013 27260688

[14] Agarwal A, Roychoudhury S, Sharma R, Gupta S, Majzoub A, Sabanegh E: Diagnostic application of oxidation-reduction potential assay for measurement of oxidative stress: clinical utility in male factor infertility. Reprod Biomed Online. 2017; 34:48-57. 10.1016/j.rbmo.2016.10.008 27839743

[15] Arafa M, Henkel R, Agarwal A, Majzoub A, Elbardisi H. Correlation of oxidation-reduction potential with hormones, semen parameters and testicular volume. Andrologia. 2019; 51:e13258. 10.1111/and.13258 30809834

[16] Garcia-Segura S, Ribas-Maynou J, Lara-Cerrillo S, Garcia-Peiró A, Castel AB, Benet J, et al. Relationship of Seminal Oxidation-Reduction Potential with Sperm DNA Integrity and pH in Idiopathic Infertile Patients. Biology (Basel). 2020; 9:262. 10.3390/biology9090262 PMC7564726 32882928

[17] Cicek OSY, Kaya G, Alyuruk B, Doger E, Girisen T, Filiz S. The association of seminal oxidation reduction potential with sperm parameters in patients with unexplained and male factor ınfertility. Int Braz J Urol. 2021; 47:112-9. 10.1590/S1677-5538.IBJU.2019.0751 PMC7712687 33047916

[18] [No Authors]. World Health Organization. (2010). WHO laboratory manual for the examination and processing of human semen, 5th ed. World Health Organization. [Internet]. Available at. <https://apps.who.int/iris/handle/10665/44261>

[19] Evenson D, Jost L. Sperm chromatin structure assay for fertility assessment. Curr Protoc Cytom. 2001; Chapter 7: Unit 7.13. 10.1002/0471142956.cy0713s13 18770725

[20] Christensen P, Sills ES, Fischer R, Naether OGJ, Walsh D, Rudolf K, et al. Impact of sperm DNA fragmentation on reproductive outcome following IVF and ICSI: a retrospective analysis of 406 cases. Presented at ESHRE 2013, Poster P-026.

[21] Evenson DP, Jost LK, Marshall D, Zinaman MJ, Clegg E, Purvis K, et al. Utility of the sperm chromatin structure assay as a diagnostic and prognostic tool in the human fertility clinic. Hum Reprod. 1999; 14:1039-49. 10.1093/humrep/14.4.1039 10221239

[22] Misell LM, Holochwost D, Boban D, Santi N, Shefi S, Hellerstein MK, et al. A stable isotope-mass spectrometric method for measuring human spermatogenesis kinetics in vivo. J Urol. 2006; 175:242-6. 10.1016/S0022-5347(05)00053-4 16406920

[23] [No Authors]. Daily Value on the New Nutrition and Supplement Facts Labels. U. S. Food & DRUG 2021. [Internet]. Available at. <https://www.fda.gov/food/new-nutrition-facts-label/daily-value-new-nutrition-and-supplement-facts-labels>

[24] Elbardisi H, Finelli R, Agarwal A, Majzoub A, Henkel R, Arafa M. Predictive value of oxidative stress testing in semen for sperm DNA fragmentation assessed by sperm chromatin dispersion test. Andrology. 2020; 8:610-7. 10.1111/andr.12743 31828966

[25] Hallak J, Teixeira TA. Oxidative Stress & Male Infertility - A necessary and conflicted indissociable marriage: How and when to call for evaluation? Int Braz J Urol. 2021; 47:686-9. 10.1590/S1677-5538.IBJU.2019.0751.1 PMC7993953 33621027

[26] Jeremias JT, Belardin LB, Okada FK, Antoniassi MP, Fraietta R, Bertolla RP, et al. Oxidative origin of sperm DNA fragmentation in the adult varicocele. Int Braz J Urol. 2021; 47:275-83. 10.1590/S1677-5538.IBJU.2019.0827 PMC7857753 33146981

[27] Pini T, Makloski R, Maruniak K, Schoolcraft WB, Katz-Jaffe MG. Mitigating the Effects of Oxidative Sperm DNA Damage. Antioxidants (Basel). 2020; 9:589. 10.3390/antiox9070589 PMC7402125 32640607

[28] Steiner AZ, Hansen KR, Barnhart KT, Cedars MI, Legro RS, Diamond MP, et al. The effect of antioxidants on male factor infertility: the Males, Antioxidants, and Infertility (MOXI) randomized clinical trial. Fertil Steril. 2020; 113:552-560.e3. 10.1016/j.fertnstert.2019.11.008 PMC7219515 32111479

[29] Smits RM, Mackenzie-Proctor R, Yazdani A, Stankiewicz MT, Jordan V, Showell MG. Antioxidants for male subfertility. Cochrane Database Syst Rev. 2019; 3:CD007411. 10.1002/14651858.CD007411.pub4 PMC6416049 30866036

[30] Esteves SC, Santi D, Simoni M. An update on clinical and surgical interventions to reduce sperm DNA fragmentation in infertile men. Andrology. 2020; 8:53-81. 10.1111/andr.12724 31692293

[31] Santi D, Spaggiari G, Simoni M. Sperm DNA fragmentation index as a promising predictive tool for male infertility diagnosis and treatment management - meta-analyses. Reprod Biomed Online. 2018; 37:315-26. 10.1016/j.rbmo.2018.06.023 30314886

[32] Roque M, Esteves SC. Effect of varicocele repair on sperm DNA fragmentation: a review. Int Urol Nephrol. 2018; 50:583-603. 10.1007/s11255-018-1839-4 29542060

[33] Lira Neto FT, Roque M, Esteves SC. Effect of varicocelectomy on sperm deoxyribonucleic acid fragmentation rates in infertile men with clinical varicocele: a systematic review and meta-analysis. Fertil Steril. 2021: S0015-0282, 00288-0. Epub ahead of print. 10.1016/j.fertnstert.2021.04.003 33985792

[34] Esteves SC, Sánchez-Martín F, Sánchez-Martín P, Schneider DT, Gosálvez J. Comparison of reproductive outcome in oligozoospermic men with high sperm DNA fragmentation undergoing intracytoplasmic sperm injection with ejaculated and testicular sperm. Fertil Steril. 2015; 104:1398-405. 10.1016/j.fertnstert.2015.08.028 26428305

[35] Jayasena CN, Sharma A, Abbara A, Luo R, White CJ, Hoskin SG, et al. Burdens and awareness of adverse self-reported lifestyle factors in men with sub-fertility: A cross-sectional study in 1149 men. Clin Endocrinol (Oxf). 2020; 93:312-21. 10.1111/cen.14213 32362009

[36] Gaskins AJ, Mendiola J, Afeiche M, Jørgensen N, Swan SH, Chavarro JE. Physical activity and television watching in relation to semen quality in young men. Br J Sports Med. 2015; 49:265-70. 10.1136/bjsports-2012-091644 PMC3868632 23380634

[37] Priskorn L, Jensen TK, Bang AK, Nordkap L, Joensen UN, Lassen TH, et al. Is Sedentary Lifestyle Associated With Testicular Function? A Cross-Sectional Study of 1,210 Men. Am J Epidemiol. 2016; 184:284-94. 10.1093/aje/kwv338 27501721

[38] Sun B, Messerlian C, Sun ZH, Duan P, Chen HG, Chen YJ, et al. Physical activity and sedentary time in relation to semen quality in healthy men screened as potential sperm donors. Hum Reprod. 2019; 34:2330-9. 10.1093/humrep/dez226 31858122

[39] Rago R, Salacone P, Caponecchia L, Sebastianelli A, Marcucci I, Calogero AE, et al. The semen quality of the mobile phone users. J Endocrinol Invest. 2013; 36:970-4. 10.3275/8996 23722985

[40] Sciorio R, Tramontano L, Esteves SC. Effects of mobile phone radiofrequency radiation on sperm quality. Zygote. 2021: 1-10. Epub ahead of print. 10.1017/S096719942100037X 34384508

[41] Hagras AM, Toraih EA, Fawzy MS. Mobile phones electromagnetic radiation and NAD+-dependent isocitrate dehydrogenase as a mitochondrial marker in asthenozoospermia. Biochim Open. 2016; 3:19-25. 10.1016/j.biopen.2016.07.003 PMC5802047 29450127

[42] Nassan FL, Jensen TK, Priskorn L, Halldorsson TI, Chavarro JE, Jørgensen N. Association of Dietary Patterns With Testicular Function in Young Danish Men. JAMA Netw Open. 2020; 3:e1921610. 10.1001/jamanetworkopen.2019.21610 PMC7043196 32083688

